# Intron-centric estimation of alternative splicing from RNA-seq data

**DOI:** 10.1093/bioinformatics/bts678

**Published:** 2012-11-21

**Authors:** Dmitri D. Pervouchine, David G. Knowles, Roderic Guigó

**Affiliations:** ^1^Centre de Regulació Genòmica (CRG) and ^2^Universitat Pompeu Fabra (UPF), 08003 Barcelona, Spain and ^3^Moscow State University, 119992 Moscow, Russia

## Abstract

**Motivation:** Novel technologies brought in unprecedented amounts of high-throughput sequencing data along with great challenges in their analysis and interpretation. The percent-spliced-in (PSI, 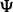) metric estimates the incidence of single-exon–skipping events and can be computed directly by counting reads that align to known or predicted splice junctions. However, the majority of human splicing events are more complex than single-exon skipping.

**Results:** In this short report, we present a framework that generalizes the 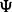 metric to arbitrary classes of splicing events. We change the view from exon centric to intron centric and split the value of 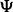 into two indices, 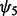 and 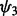, measuring the rate of splicing at the 5′ and 3′ end of the intron, respectively. The advantage of having two separate indices is that they deconvolute two distinct elementary acts of the splicing reaction. The completeness of splicing index is decomposed in a similar way. This framework is implemented as bam2ssj, a BAM-file–processing pipeline for strand-specific counting of reads that align to splice junctions or overlap with splice sites. It can be used as a consistent protocol for quantifying splice junctions from RNA-seq data because no such standard procedure currently exists.

**Availability:** The C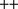 code of bam2ssj is open source and is available at https://github.com/pervouchine/bam2ssj

**Contact:** dp@crg.eu

## 1 INTRODUCTION

One major challenge in the analysis of high-throughput RNA sequencing data is to disentangle relative abundances of alternatively spliced transcripts. Many existing quantification methods do so by using considerations of likelihood, parsimony and optimality to obtain a consolidated view of cDNA fragments that map to a given transcriptional unit (Katz *et al.*, 2010; Montgomery *et al.*, 2010; Trapnell *et al.*, 2012). The advantage of such integrative approaches is that they provide robust estimators for transcript abundance by reducing sampling errors, as they effectively consider samples of larger size. In contrast, because all the reads from the same transcriptional unit are combined into one master model, there is no guarantee that the inclusion or exclusion of a specific exon is estimated independently of co-occurring splicing events (Katz *et al.*, 2010; Pan *et al.*, 2008).

**Fig. 1.**
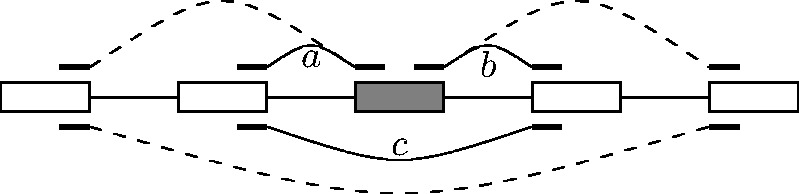
The percent-spliced-in (PSI, 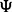) metric is defined as the number of reads supporting exon inclusion (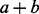) as the fraction of the combined number of reads supporting inclusion and exclusion (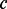). The exon of interest is shown in gray. Only reads that span to the adjacent exons (solid arcs) account for Equation (1)

The quantification of alternatively spliced isoforms based on the 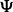 metric captures more accurately the local information related to splicing of each particular exon (Katz *et al.*, 2010). We follow Kakaradov *et al.* (2012) in considering only the reads that align to splice junctions (Fig. 1) and ignoring the reads that align to exon bodies (position-specific read counts are not considered). 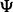 is defined as (1)
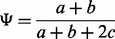
 where the factor of two in the denominator accounts for the fact that there are twice as many mappable positions for reads supporting exon inclusion as exon exclusion. Equation (1) defines an unbiased estimator for the fraction of mRNAs that represent the inclusion isoform under the assumption that splice-junction reads are distributed evenly. 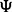 can also be derived from the expression values of whole isoforms, for instance, as the abundance of the inclusion isoform as the fraction of the total abundance. However, the non-uniform read coverage not only between but also within transcripts makes such estimates generally detrimental (Kakaradov *et al.*, 2012).

The 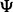 metric can be generalized beyond the class of single-exon–skipping events by counting inclusion and exclusion reads regardless of exon adjacency (Fig. 1, dashed arcs). Although this definition helps to reduce the undercoverage bias by taking into account splice junctions that are not present in the reference annotation, it often assigns misleading values to 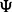 metric, for instance, in the case of multiple-exon skipping, where the amount of support for exon exclusion does not reflect the true splicing rate of each individual intron.

## 2 APPROACH

**Fig. 2.**
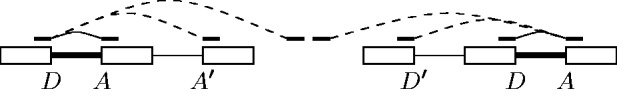
Left: the 5′-splicing index, 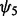, is the number of reads supporting the splicing event from 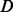 to 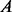 relative to the combined number of reads supporting splicing from 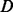 to any acceptor site 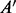. Right: the 3′-splicing index, 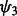, is the number of reads supporting the splicing event from 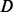 to 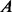 relative to the combined number of reads supporting splicing from any donor site 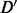 to 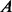. The intron of interest is drawn thick

In this work, we change the view from exon centric to intron centric. Each intron is defined uniquely by the combination of its 5′-splice site (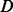, donor) and 3′-splice site (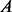, acceptor). Denote by 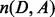 the number of reads aligning to the splice junction spanning from 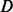 to 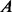 (Fig. 2) and define (2)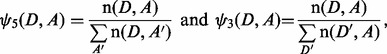 where 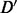 and 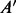 run over all donor and acceptor sites, respectively, within the given genomic annotation set. Because 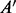 could be 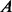 and 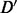 could be 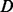, both 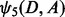 and 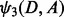 are real numbers from 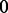 to 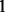. The value of 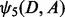 can be regarded as an estimator for the conditional probability of splicing from 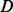 to 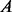, i.e. the fraction of transcripts in which the intron 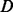 to 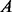 is spliced, relative to the number of transcripts in which 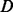 is used as a splice site. Similarly, 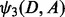 is the relative frequency of 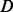-to-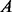 splicing with respect to the splicing events in which 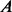 is used.

In the particular case of single-exon skipping (Fig. 1), the values of 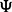, 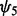 and 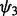 are related as follows. Denote the upstream and downstream introns of the highlighted exon by 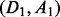 and 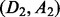, respectively. Let 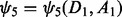 and 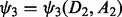. Then, 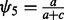, 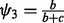 and 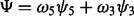, where 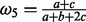 and 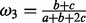. Assuming uniform read coverage across the gene (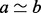), we get 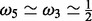 and, therefore, (3)
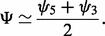
 That is, in the particular case of single-exon skipping, the value of 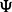 is equal to the average of 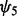 and 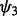 given that the read coverage is reasonably uniform. If *a* and 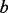 differ significantly, the contribution of 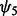 and 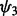 to 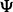 is given by the weight factors 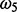 and 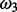.

Similarly, the completeness of splicing index (Tilgner *et al.*, 2012) is split into two indices, 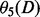 and 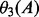, where (4)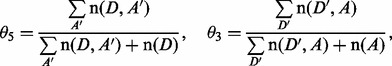 and 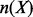 denotes the number of genomic reads (reads mapped uniquely to the genomic sequence) overlapping the splice site 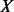. Note that 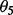 depends only on 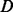 and 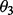 depends only on 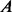. The values of 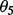 and 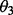 are unbiased estimators for the absolute frequency of splice site usage, i.e. the proportion of transcripts in which 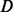 (or 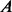) is used as a splice site, among all transcripts containing the splice site 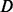 (or 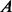).

## 3 METHODS

To compute 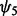, 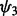, 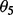 and 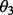 for a given donor–acceptor pair, one needs to know five integers, 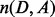, 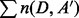, 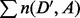, 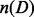 and 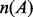, of which only the first one depends on both 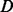 and 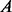, while the rest have a single argument. We developed bam2ssj, a pipeline for counting these five integers directly from BAM input. bam2ssj is implemented in C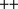 and depends on SAMtools (Li *et al.*, 2009). The input consists of (i) a sorted BAM file containing reads that align uniquely to the genome or to splice junctions and (ii) a sorted GTF file containing the coordinates of exon boundaries. Each time the CIGAR string (Li *et al.*, 2009) contains 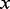
M
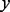
N
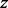
M, 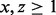, the counter corresponding to the splice junction defined by 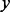
N is incremented. One mapped read may span several splice junctions and increment several counters. If the CIGAR string does not contain the 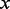
M
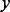
N
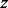
M pattern, the read is classified as genomic and increments 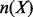 for every splice site 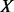 it overlaps. Position-specific counts (Kakaradov *et al.*, 2012) are implemented as a stand-alone utility that is not included in the current distribution. Importantly, bam2ssj counts reads that align to splice junctions in a strand-specific way, i.e. 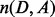, 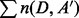, 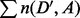, 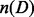 and 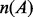 are reported for the correct (annotated) and incorrect (opposite to annotated) strand. We leave further processing of these counts by Equations (2)–(4) to the user.

## 4 RESULTS AND DISCUSSION

We validated bam2ssj by counting reads aligning to splice junctions in the whole-cell polyadenylated fraction of Cold Spring Harbor Long RNA-seq data (http://genome.ucsc.edu/ENCODE/). In total, 8 558 231 343 mapped reads were analyzed in 404 min (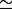
350 000 reads/sec). 1 184 553 724 reads align to splice junctions, of which 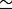
1% align to the opposite strand. 1 699 718 327 reads overlap annotated splice junctions, of which 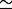
5% map to the opposite strand. The values of 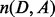 coincide with those reported by ENCODE in 98.9% of cases (1 163 251 008 reads); all discrepancies were due to the ambiguity of CIGAR translation in the mapper’s output. Because RNA-seq data are increasingly processed into the compact BAM form, we propose that bam2ssj be used as a standard operating procedure for counting splice junction reads.

*Funding*: Grants BIO2011-26205 and CSD2007-00050 Consolider, Ministerio de Educación y Ciencia (Spain).

*Conflict of Interest*: none declared.
